# YIPFα1A expression is regulated by multilayered molecular mechanisms

**DOI:** 10.1002/2211-5463.70244

**Published:** 2026-04-06

**Authors:** Tokio Takaji, Yurika Nakanishi, Nobuhiro Nakamura

**Affiliations:** ^1^ Division of Life Sciences Graduate School of Kyoto Sangyo University Japan; ^2^ CRIA, Kyoto University Japan

**Keywords:** 3′ untranslated region, codon adaptation index, Golgi, mRNA stability, translation, transmembrane protein

## Abstract

Yip domain family (YIPF) proteins are five‐pass transmembrane proteins that localize primarily to the Golgi apparatus. These proteins assemble into higher‐order complexes with each *α*‐subunit pairing specifically with a *β*‐subunit to form a dimer which then assemble into complexes with two to four dimers. Notably, *β*‐subunit expression depends on the corresponding *α*‐subunit partner, and conventional transient overexpression of *α*‐subunits has been extremely inefficient, hindering deeper analysis of YIPF complexes. To identify the cause of poor exogenous expression, we examined YIPF gene features and found two properties correlated with low expression: (i) rare‐codon enrichment in the CDS and (ii) extended 3′ UTRs. Experimental analyses focusing on YIPFα1A revealed that rare‐codon enrichment suppresses expression mainly at the mRNA level, consistent with translation‐coupled mRNA decay, whereas inclusion of the native 3′ UTR enhances expression by increasing mRNA abundance. Deletion mapping further showed that a proximal 3′ UTR segment (51–150) is necessary and sufficient for mRNA stabilization, thereby elevating both mRNA and protein levels. Conversely, a distal 3′ UTR fragment (1116–2230) increased mRNA but not protein levels, suggesting translational repression resulting in a reduced protein‐to‐mRNA ratio. Together, these findings explain the discrepancy between endogenous and exogenous YIPFα1A expression and propose a multilayered regulatory model in which rare codons decrease mRNA, the proximal 3′ UTR stabilizes mRNA, and the distal 3′ UTR reduces translation.

**Impact statement**

Our work advances YIPF biology and identifies post‑transcriptional mechanisms governing multi‑pass membrane proteins. We show rare‑codon and 3′ UTR‑based control of trafficking proteins—an area largely unexplored—and introduce a new paradigm for membrane‑traffic regulation that will guide future studies of complex assembly, localization, and homeostasis.

## Graphical abstract

YIPFα1A, a five‐pass Golgi protein, is regulated at multiple layers. (1) Rare‐codon enrichment drives translation‐coupled mRNA decay. (2) A proximal 3′‐UTR element stabilizes mRNA. (3) A distal 3′‐UTR element included by alternate poly(A) site usage represses translation, which can be overridden by the proximal 3′‐UTR element. (4) Translation inhibition induces endogenous YIPFα1A expression.

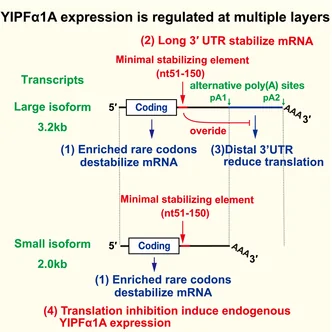

**Abbreviations**
ANOVA: analysis of variance
CAI: codon adaptation index
CDS: coding sequence
CMV: cytomegalovirus
GAPDH: glyceraldehyde‐3‐phosphate dehydrogenase
HA: hemagglutinin
kb: kilobase
LSD: least significant difference
nt: nucleotide
poly(A) signal: polyadenylation signal
SEM: standard error of the mean
SV40: simian virus 40
UTR: untranslated region
YIPF: Yip domain family

The Golgi apparatus is a central organelle in the secretory pathway. Newly synthesized secretory and transmembrane proteins in the endoplasmic reticulum (ER) are transported to the Golgi apparatus, where they undergo further processing and sorting, before being directed to their final destinations, such as the lysosome, plasma membrane, or extracellular space. The primary functions of the Golgi apparatus include the synthesis and modification of glycan chains, sulfation of amino acid side chains and glycans, and proteolytic cleavage required for the maturation of transit proteins [1, 2, 3, 4].

In vertebrates, including mammals, the Golgi apparatus exhibits a characteristic stacked cisternal organization [5]. The enzymes responsible for protein processing are distributed in a polarized manner within these cisternae: early acting enzymes are concentrated on the *cis* side (entry), whereas late‐acting enzymes are enriched on the *trans* side (exit) [6, 7]. Over the past half‐century, the molecular mechanisms that regulate vesicular transport from the ER, through the Golgi apparatus, and toward final destinations have been extensively investigated [8]. This body of work was recognized with the Nobel Prize in Physiology or Medicine in 2013 [9, 10].

In parallel, the mechanisms underlying the localization of Golgi membrane proteins—particularly those involved in protein glycosylation—have also been investigated, yielding important insights [9, 10]. However, the precise molecular mechanisms that maintain the polarized distribution of Golgi‐resident proteins remain unclear [6, 7]. Our research has focused on elucidating the molecular principles that govern Golgi structure and protein localization, specifically through the interactions between peripheral membrane proteins—such as GM130 and GRASP65—and integral transmembrane proteins [11, 12, 13, 14, 15].

During the course of these studies, we became interested in a family of multi‐pass transmembrane proteins, now referred to as YIP domain family (YIPF) proteins, which may serve as an interface between peripheral and integral Golgi membrane proteins [16]. YIPF proteins constitute a family of five‐pass transmembrane proteins that localize primarily to the Golgi apparatus and are conserved across the eukaryotic kingdom [16]. Yip1p and Yif1p, the founding members of this family, were originally identified in budding yeast *Saccharomyces cerevisiae* [17, 18]. These proteins are essential for cell growth and have been proposed to promote the fusion of ER‐derived transport vesicles with the Golgi apparatus in cooperation with Rab/Ypt family small GTPases [17, 18]. Subsequent studies further suggested their involvement in vesicle budding at the ER; however, whether they directly participate in vesicle formation or instead confer fusion competency to the target membranes remains a matter of debate [19, 20, 21]. In addition to Yip1p and Yif1p, budding yeast also possesses two nonessential paralogues, Yip4p and Yip5p whose biological functions are still unknown [17, 18, 22, 23].

Over the past two decades, we have uncovered several fundamental molecular characteristics of YIPF proteins. (1) Nine family members are present in humans and other mammals. These proteins fall into two subgroups, represented by the yeast proteins Yip1p and Yif1p. One member from each subgroup forms a dimer in a specific pairing, and two to four dimers subsequently assemble into higher‐order complexes [24, 25, 26]. (2) These dimers constitute three distinct complexes with partially overlapping distributions: the YIPF1 complex localizes mainly to the ER‐Golgi intermediate compartment (ERGIC), the YIPF2 complex to the *cis*‐Golgi, and the YIPF3 complex to the *trans*‐Golgi. Based on these features, we previously classified the Yip1p subgroup as *α*‐subunits and the Yif1p subgroup as *β*‐subunits, renaming each YIPF member according to its complex localization and subgroup identity [16]. (3) Expression of each *β*‐subunit depends on the presence of its *α*‐subunit partner, as knockdown of an *α*‐subunit reduces the corresponding *β*‐subunit protein level. (4) The YIPF1 and YIPF2 complexes contribute to maintaining Golgi structure, whereas the YIPF3 complex functions in proper glycosylation [24, 25, 26].

During our studies, we observed that most YIPF *α*‐subunits were poorly expressed in standard transient expression systems using conventional plasmid expression vectors containing only the coding sequence (CDS). This low expression occurred even under strong promoters such as the cytomegalovirus (CMV) promoter and the human elongation factor 1*α*‐subunit (hEF‐1α) promoter, thereby hindering further analysis of YIPF complexes. We therefore sought to determine the underlying mechanism for the poor expression of YIPF *α*‐subunits. Careful examination of YIPF gene structures revealed two common features among the poorly expressed YIPF *α*‐subunits: an enrichment of rare codons within their CDSs and the presence of extended 3′ untranslated regions (UTRs) in their mRNAs. Experimental analyses demonstrated that both features significantly influence YIPFα1A expression. Rare‐codon enrichment markedly suppressed YIPFα1A protein production, whereas the YIPFα1A 3′ UTR enhanced protein expression. Deletion analysis further revealed that a proximal region of the 3′ UTR immediately downstream of the CDS is necessary and sufficient to increase YIPFα1A expression by stabilizing its mRNA. The implications of these regulatory mechanisms at both the mRNA stability and translational levels are discussed.

## Methods

### Reagents and cell culture

All reagents were biochemical or molecular biology grade (Nacalai Tesque, Kyoto, Japan or FUJIFILM Wako Pure Chemical Corp., Osaka, Japan). Restriction enzymes were purchased (New England Biolabs, Ipswich, MA, USA, TAKARA Bio Inc., Shiga, Japan or TOYOBO Co. Ltd., Osaka, Japan). Authenticated and mycoplasma‐free HEK293 cells (RRID: CVCL_0045) were obtained from the American Type Culture Collection (ATCC; CRL‐1573). Cells were cultured in high‐glucose Dulbecco's Modified Eagle's Medium (DMEM; FUJIFILM Wako) supplemented with 10% fetal bovine serum (Sigma‐Aldrich, St. Louis, MO, USA) and maintained at 37 °C in a humidified atmosphere containing 5% CO_2_. The master stock obtained from ATCC was expanded for several weeks to generate liquid nitrogen stocks. Thawed cells were used for experiments within 3 months of culture.

### cDNA and plasmid construction

We described cloning of YIPF protein cDNAs previously [27], except for YIPFα1B, which was purchased (Sino Biological Inc., Beijing, China). The cDNA sequence of the 3′ UTR of YIPFα1A and GAPDH was cloned by RT‐PCR using a laboratory‐made cDNA library. The poly(A) RNA was purified from HEK293 cells using MagExtractor RNA (TOYOBO) and Oligotex‐dT30 <Super> mRNA Purification Kit (TAKARA Bio). It was reverse transcribed using PrimeScript II 1st Strand cDNA Synthesis Kit (TAKARA Bio) following the manufacturer's protocol. PCR was performed using deduced primers to amplify from the end of the CDS to the 3′ end of the longest cDNA sequence found in the database, excluding the poly(A) tail (YIPF1αA: NM_001024947, 2230 bp; GAPDH: BC083511, 200 bp). The sequence completely matched the database sequence. Native 3′ UTR sequence was reproduced by attaching to the YIPFα1A CDS using In‐Fusion cloning (TAKARA Bio) following the manufacturer's protocol. Full‐length and deletion fragments of the 3′ UTR were produced by PCR using a pair of primers containing appropriate restriction sites (AscI, SbfI). Expression plasmid vectors were constructed by replacing the EGFP CDS on pEGFP‐C1 (TAKARA Bio) with YIPF CDSs, attaching an epitope tag at the N terminus and restriction sites at N‐ (NheI) and C terminus (XhoI) for ease of cloning. Restriction sites (AscI and SbfI) were also introduced into the expression plasmid vectors for inserting various fragments of the 3′ UTR at the 3′ end of the CDS (TCTAGA GGCGCGCC ACAT CCTGCAGG CTGATCATAATC), the 5′ of the CMV promoter (GATTCTGT GGCGCGCC ACAT CCTGCAGG ATAACCGTAT) or the 3′ of the SV40 poly(A) signal (GTATCTTAACGCG GGCGCGCC ACAT CCTGCAGG CGCGTAAA). Plasmid vector cloning was performed using QuikChange Site‐Directed Mutagenesis (Agilent Technologies Inc., Santa Clara, CA, USA), In‐Fusion HD cloning, or conventional restriction site cloning following the manufacturer's protocol. N‐terminal HA‐tag (MYPYDVPDY) or HiBiT‐tag with a linker sequence (MVSGWRLFKKIS GGGG; Promega Corp., Madison, WI, USA) was attached in‐frame with CDSs, interrupted by the NheI site (GCTAGC; AS). The original C‐terminal stop codon was removed and replaced with XhoI and XbaI sites (CTCGAGCTCTAGA; LEL) for previously described C‐terminal tagging studies [27]. For transfection experiments, plasmids were purified using the Plasmid Plus Midi Kit (QIAGEN GmbH, Hilden, Germany). Codon optimization was performed by PCR using appropriate primers. Briefly, the CDS was divided into seven segments with overlaps of approximately 20 base pairs. Each segment was PCR‐amplified using appropriate primers containing designed mutations (Table 2). The segments were joined by extension PCR, and the final fused product was cloned using terminal restriction sites (NheI and XhoI) and sequence verified.

### Transfection and cell harvest

Cells were seeded 16 h before transfection (2.0 × 10^5^ cells/3.5 cm dish for protein analysis, 4.0 × 10^5^ cells/5.0 cm dish for RNA analysis). Transfection was performed using FuGENE 6 (Promega) at 1 μg DNA, 3 μL reagent ratio for protein analysis, doubled for RNA analysis, according to the manufacturer's protocol. Twenty‐four hours after the transfection, cells were scraped from the dish using a cell scraper (Sumitomo Bakelite Co., Ltd., Tokyo, Japan) in the presence of 1 mL growth medium on ice. The cell suspension was recovered in a 1.5‐mL centrifuge tube, spun for 5 s at maximum speed at 4 °C to pellet the cells, and the supernatant was removed. The cells were washed by adding 1 mL ice‐cold Dulbecco's PBS and spun for 5 s at maximum speed at 4 °C three times for protein extraction or once for RNA extraction. For protein analysis, 50 μL of 4% SDS, 0.05 M Tris–HCl (pH6.7) solution was added, vortex‐mixed to detach cell pellet and sonicated using BIORUPTOR (Sonicbio Co., Ltd., Kanagawa, Japan) until solution was no longer viscous (5–10 min with 20‐s intervals every 20 s in ice‐cold water bath). The protein concentration of the cell lysate was measured using the Pierce BCA Protein Assay Kit (Thermo Fisher Scientific Inc., Rockford, IL, USA). An equal volume of 0.02% BPB, 0.05 M Tris–HCl (pH6.7), 0.4 M sucrose, 0.2 M dithiothreitol was added, and the protein concentration was adjusted to 1.0 μg·μL^−1^ using 1 × SDS/PAGE sample buffer (2% SDS, 0.01% BPB, 0.025 M Tris–HCl (pH6.7), 0.2 M Sucrose, 0.1 M dithiothreitol). Total RNA was extracted using the MagExtractor RNA (TOYOBO) or the RNeasy Plus Mini Kit (QIAGEN) according to the manufacturer's protocol. RNA concentration was measured using a BioSpectrometer equipped with a μCuvette G1.0 (Eppendorf Vertrieb Deutschland GmbH, Wesseling‐Berzdorf, Germany).

### Western blotting

This was performed as described previously, using an anti‐YIPFα1A (YIPF5) antibody and anti‐HA antibody (16B12; Covance Research Products, Inc., Berkeley, CA, USA) [26]. A peroxidase‐conjugated anti‐mouse and anti‐rabbit IgG (Jackson ImmunoResearch Labs. Inc., West Grove, PA, USA) and Immobilon Western Chemiluminescent HRP Substrate (EMD Millipore Corp., Burlington, MA, USA) were used for detection. Detection of HiBiT tag was performed using the Nano‐Glo HiBiT Blotting System (Promega), according to the manufacturer's protocol.

### Northern blotting

This was performed using classical agarose gel electrophoresis and capillary transfer by downward flow [28]. Briefly, 1 μg of total RNA was loaded along with 2 μL of Prestain Marker for RNA High (BioDynamics Laboratory Inc., Tokyo, Japan) onto a 1% agarose gel containing formaldehyde and 1× MOPS electrophoresis buffer and run at 100 V for 50 min using a Mupid‐2× (Mupid Co., Ltd., Tokyo, Japan). The RNA was transferred onto a positively charged nylon membrane (Roche Diagnostics GmbH, Mannheim, Germany) for 6 h using alkaline transfer buffer. The membrane was UV‐irradiated for 2 min and subjected to hybridization and detection using a DIG‐labeled probe, DIG Easy Hyb, DIG Wash and Block Buffer Set and Anti‐Digoxigenin‐AP Fab fragments (Roche Diagnostics), following the manufacturer's protocol.

### RNA probe synthesis

RNA probes were synthesized using DIG RNA Labeling Kit (Roche Diagnostics) with PCR‐amplified templates. As templates for PCR, plasmids containing the HA‐tag only, HA‐tagged YIPFα1A CDS and human *β*‐actin CDS were constructed by inserting them between HindIII (AAGCTT CCACC ATG‐HA‐tag‐CDS) and XbaI (CDS‐CTCGAG C TCTAGA; LEL and stop codon) sites of pcDNA3 (Invitrogen, Thermo Fisher Scientific, Waltham, MA, USA). The YIPFα1A CDS with the HA tag, HA tag only (TAATACGACTCACTATAGGG AGACCCAAGCTTCCACC ATGTACCCCTACGACGTGCCCGACTACGCT AGAGGGCCCTATT CTATAGTGTCACCTAAAT; under lines indicate T7, HA tag, and SP6), and the target sequences were amplified by PCR using T7 and SP6 primers, and used for probe synthesis with SP6 primer.

### Chemiluminescence image detection and quantitation

Chemiluminescent images were taken using the ImageQuant LAS4000 mini (GE Healthcare Technologies Inc., Chicago, IL, USA). Quantitation of images was performed using ImageJ [29] with TIFF‐formatted files exported from the image analyzer. For the comparison of signal strength of bands from western blotting and northern blotting, each band of interest was manually selected using a fixed‐size rectangle (areas are indicated in figures), and the mean grayscale intensity was recorded. The grayscale intensity of each band was normalized by subtracting the background, which was the mean grayscale intensity of three background areas. To calculate the mean across experiments, the ratio of the grayscale intensity of each band to the sum of grayscale intensities of all bands was calculated in each experiment. Then, the mean, SEM of the ratio were calculated for each band, and these were further divided by the mean value of the standard to calculate the relative ratio. For the quantitation of the ratio (%) of differently sized products in northern blotting, the image was placed horizontally, and the lane of interest was manually selected using a rectangle. Then, a profile of grayscale intensity was plotted, and the grayscale intensity values at each location on the blot were exported to an Excel file using the Plot Profile command. By consulting the image, the boundaries of main, sub and degradation areas were manually determined and indicated in the figure. The grayscale intensities of each area were summed, and the background was subtracted. The value of each area was divided by the total value of the three areas to calculate the ratio (%). For the calculation of the fold increase, the fold increase values were calculated for each experiment, and those values were used to calculate the mean and SEM. Statistical analysis was performed using Statmate V (Atms Co. Ltd., Tokyo, Japan).

### qPCR

Total RNA extracted from HEK293 cells was used for quantitative PCR (qPCR) analysis. Reverse transcription was performed using the PrimeScript RT Reagent Kit with gDNA Eraser (Perfect Real Time), and qPCR was carried out with TB Green Premix Ex Taq II (Tli RNaseH Plus) (TAKARA Bio), following the manufacturer's instructions. Primers were designed according to the recommended guidelines and verified to produce a single, specific amplicon. The resulting product sequences were further confirmed. The following primer sets were used: YIPFα1A (forward: tgtccctccagacatgatgc, reverse: aaggctgaggtgaagctgga), GAPDH (forward: gcgagatccctccaaaatca, reverse: aaatgagccccagccttctc), and *c‐myc* (forward: cccggagttggaaaacaatg, gcttttgctcctctgcttgg).

## Results

### Differential expression levels among YIPF family members

**Fig. 1:**
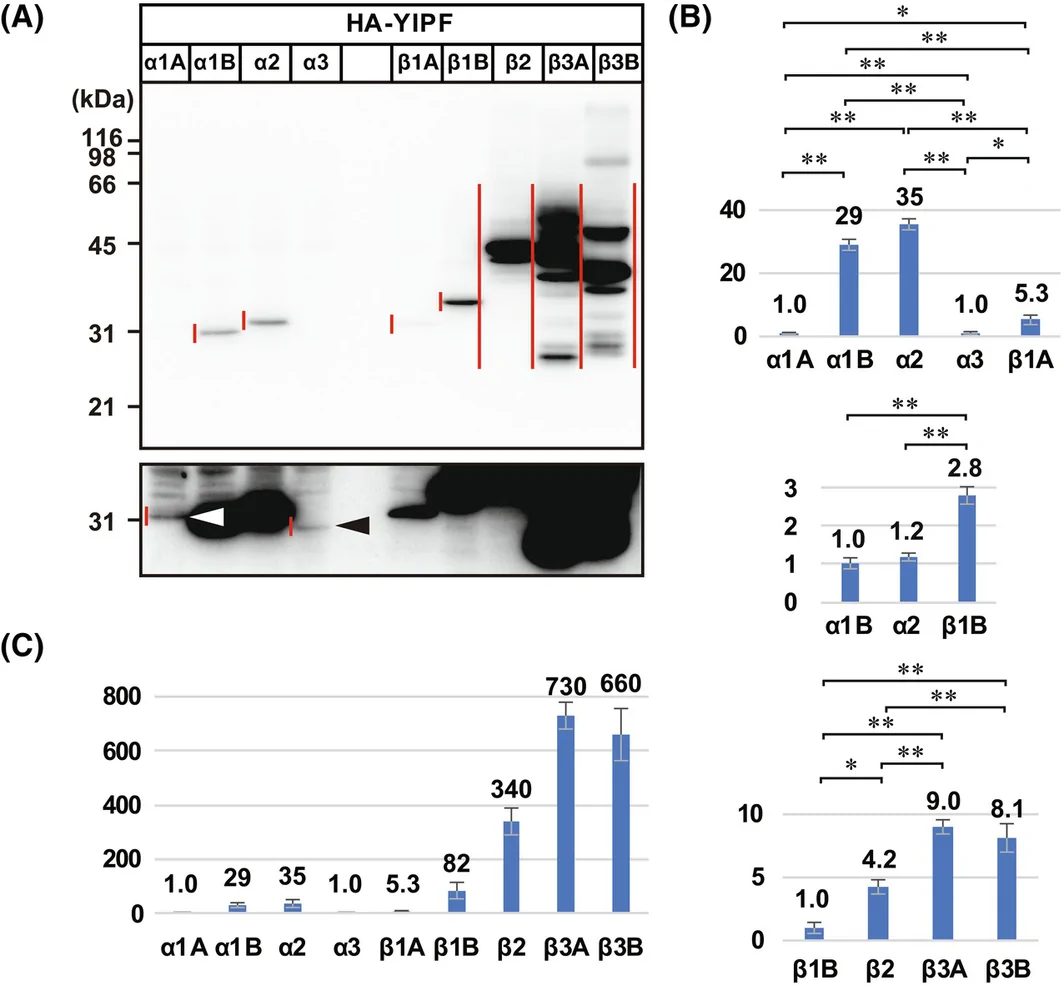
Exogenous expression efficiency of Yip domain family (YIPF) proteins. HEK293 cells were transfected with expression plasmids encoding N‐terminally hemagglutinin (HA)‐tagged YIPF proteins and analyzed by western blotting 24 h post‐transfection using an anti‐HA antibody. (A) Representative western blot showing equal protein loading (10 μg per lane) from three independent experiments. Molecular weight markers are shown on the left. The lower panel shows a longer exposure of the upper panel. White and black arrowheads indicate weak signals for YIPFα1A and YIPFα3, respectively. Red lines delineate regions used for quantitation. (B) Quantitative analysis and statistical comparison of expression ratios within three independent sets. Statistical significance was assessed by one‐way ANOVA followed by Fisher's least significant difference (LSD) test. (*P < 0.05, **P < 0.01, no mark: not significant). (C) Integrated results from (B). Values from the middle panel in (B) were multiplied by the corresponding YIPFα1B value from the upper panel, values from the lower panel were further multiplied by the YIPFβ1B value from the middle panel (already normalized to YIPFα1B). The standard propagation of uncertainty formula was applied for the calculation of standard error of the mean (SEM). Data are presented as mean with error bars indicating the SEM; *n* = 3. Statistics: one‐way ANOVA with Fisher's least significant difference (LSD) test. (*P < 0.05, **P < 0.01, no mark: not significant).

To initiate our analyses, we compared the exogenous expression levels of all YIPF family proteins by inserting their CDSs into CMV promoter‐driven expression plasmids containing an N‐terminal HA epitope tag. As shown in Fig. 1A, all YIPF proteins were successfully expressed in HEK293 cells; however, their expression levels varied markedly across YIPF proteins. To assess the significance of these differences, quantitative analysis of protein expression was performed. Because the dynamic range of expression was too large to allow direct comparison of all family members in a single set, the dataset was divided into three subsets and expression levels were analyzed as ratio values (Fig. 1B).

To reduce background signal interference, a narrow quantitation region was selected for proteins with weaker expression (YIPFα1A, YIPFα1B, YIPFα2, YIPFα3, YIPFβ1A, and YIPFβ1B; regions indicated by red lines on the left side of lanes). For proteins with stronger expression, a broader region was selected to capture post‐translationally modified forms (YIPFβ1B, YIPFβ2, YIPFβ3A, YIPFβ3B; regions indicated by red lines on the right side of lanes). Statistical analysis revealed significant differences in expression between YIPF proteins, with the exceptions of YIPFα1B versus YIPFα2 and YIPFβ3A versus YIPFβ3B (Fig. 1B).

To integrate the results across the three subsets, YIPFα1B and YIPFβ1B were used as internal reference points (Fig. 1C). The integrated analysis revealed a consistent pattern: *α*‐subunits exhibited uniformly low expression levels, whereas *β*‐subunits were expressed at generally higher levels (except for YIPFβ1A and YIPFβ1B). Notably, YIPFα1A and YIPFα3 showed extremely low expression—less than 1/700th of YIPFβ3A or YIPFβ3B. Because all constructs were expressed under identical promoter and terminator conditions, these differences in protein expression levels can be attributed to intrinsic properties of the corresponding CDSs.

### Genetic characteristics of YIPF proteins

To investigate why certain YIPF proteins are poorly expressed, we conducted a detailed analysis of the genetic features of the YIPF family. Two characteristics were consistently observed among YIPF *α*‐subunits (Table 1). The first was a lower codon adaptation index (CAI) [30]. As summarized in Table 1, most *α*‐subunits (YIPFα1A, YIPFα2, and YIPFα3) exhibited CAI values below 0.7, whereas all *β*‐subunits showed CAI values above this threshold. A lower CAI indicates an enrichment of rare codons, which is predicted to reduce the translational efficiency of the corresponding CDSs [31].

**Table 1:** mRNA information of YIPF family proteins.

YIPF	Relative expression^a^	Northern blotting (bp)^b^	RefSeq cDNA (bp)	3′ UTR (bp)	CAI	RefSeq
α1A	1	3600	3395	2235	0.67	NM_001024947
α1B	30	ND	1170	326	0.72	NM_182592
α2	30	2300	2084	1175	0.65	XM_011533125
α3	1	7500	6010	5283	0.69	NM_173834
β1A	6	1300	1097	57	0.82	NM_020470
β1B	70	5300	2690	1794	0.79	NM_001039672
β2	300	3000	1507	335	0.80	NM_015388
β3A	600	2300	1821	546	0.73	NM_018982
β3B	600	2400	2111	1001	0.84	NM_001321439

^a^ Relative values for YIPFα1A

^b^ Shakoori, A. *et al. Biochem Biophys Res Commun* 312, 850–857 (2003).

The second common feature of *α*‐subunits was the presence of extended 3′ UTRs in their mRNAs (Table 1). The 3′ UTR is known to regulate protein expression by stabilizing or destabilizing the transcripts and/or modulating translation efficiency [32, 33, 34]. In addition, the 3′ UTR can direct transcript localization to specific subcellular regions, thereby influencing the site of translation and/or facilitating interactions between nascent proteins and other cellular components—processes that ultimately affect translation efficiency and protein function [32, 33, 34]. Because the 3′ UTR was absent from the expression constructs used in our initial exogenous expression experiments (Fig. 1), its omission may have contributed to the reduced protein expression observed for YIPF *α*‐subunits.

Given these findings, we focused on YIPFα1A to evaluate the individual contributions of rare‐codon enrichment and the extended 3′ UTR to protein expression.

### Codon usage affects YIPFα1A expression

The CAI of wild‐type (WT) YIPFα1A is 0.67. Notably, 43 codons in the YIPFα1A CDS are classified as rare (< 16%) based on the human codon usage table [30, 35]. To assess the effect of rare‐codon enrichment on protein expression, all 43 rare codons were replaced with higher‐frequency codons to generate a codon‐optimized (Op) mutant (Table 2, CAI = 0.84). Both the WT and Op mutant constructs were tagged at the N terminus with an HA epitope to distinguish exogenous proteins from endogenous proteins, and they were expressed in HEK293 cells as described above.

**Table 2:** Codon optimization of YIPFα1A.

No.	WT^a^	AA	Opt^a^	No.	WT^a^	AA	Opt^a^	No.	WT^a^	AA	Opt^a^	No.	WT^a^	AA	Opt^a^
2	TCA	S	AGC	108	CTA	L	CTG	156	AGT	S	AGC	194	CTT	L	CTG
7	TTA	L	CTG	110	GTA	V	GTG	161	CTA	L	CTG	203	TTG	L	CTG
9	ACG	T	ACC	111	TTA	L	CTG	166	TTA	L	CTG	207	GTA	V	GTG
15	AGT	S	AGC	113	CCG	P	CCC	167	TTA	L	CTG	220	AGT	S	AGC
22	TCA	S	AGC	114	TTA	L	CTG	169	TTA	L	CTG	231	TTA	L	CTG
31	AGT	S	AGC	116	GTA	V	GTG	171	AGT	S	AGC	238	CTT	L	CTG
45	TCG	S	AGC	127	TTG	L	CTG	176	TCA	S	AGC	239	TTA	L	CTG
77	TCA	S	AGC	135	CTT	L	CTG	182	AGT	S	AGC	240	GTA	V	GTG
92	TTA	L	CTG	141	TTG	L	CTG	184	CCT	L	CTG	246	TTG	L	CTG
93	TTA	L	CTG	142	CTA	L	CTG	188	CTT	L	CTG	247	TTA	L	CTG
96	TTA	L	CTG	152	GTA	V	GTG	193	CTA	L	CTG				

^a^ WT: CAI = 0.67, Opt: CAI = 0.82.

**Fig. 2:**
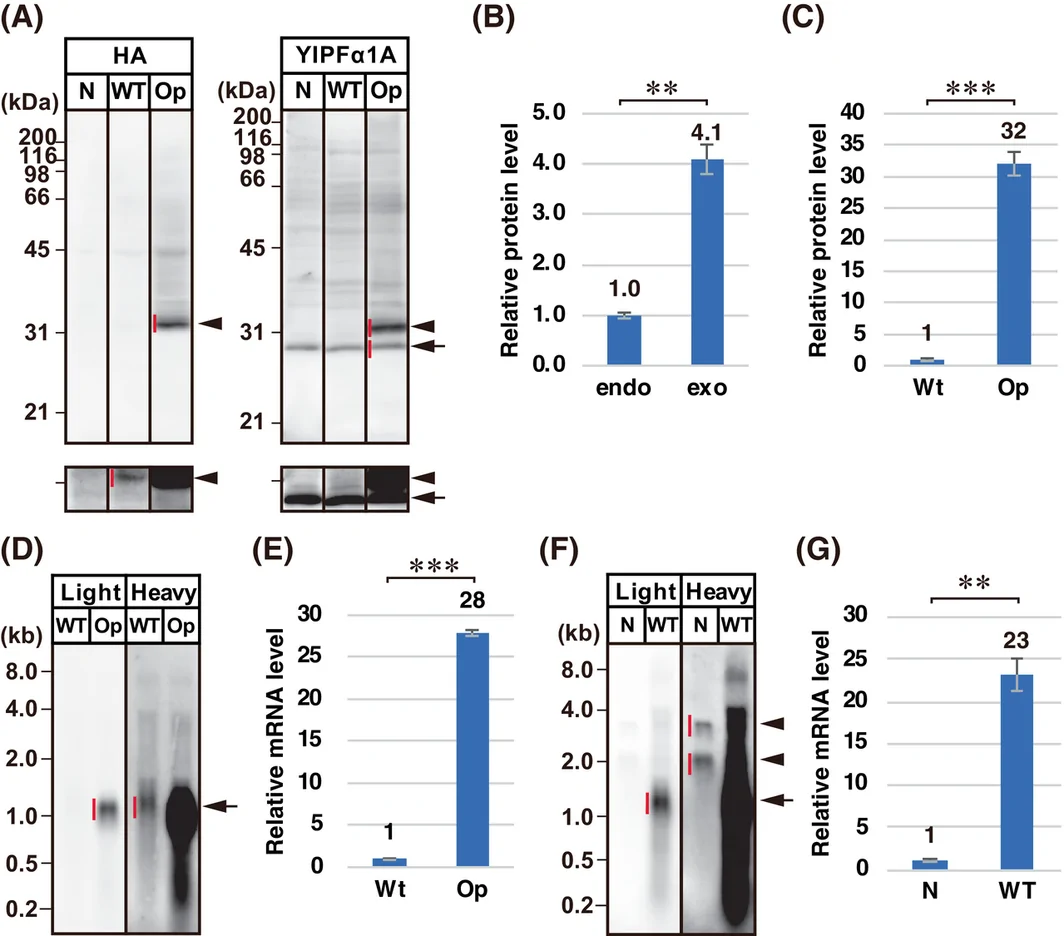
Codon optimization enhances YIPFα1A expression. HEK293 cells were transfected with N‐terminally hemagglutinin (HA)‐tagged wild‐type (WT) or codon‐optimized (Op) YIPFα1A constructs and analyzed after 24 h by western blotting (A–C) or northern blotting (D–G). N: nontransfected control. Molecular weight (Western) and size (northern) markers are shown on the left. Red lines indicate regions used for quantitation. Data are presented as mean with error bars indicating the standard error of the mean (SEM); *n* = 3. (A) Western blotting probed with anti‐HA (left) or anti‐YIPFα1A (right). Representative images from three experiments; lower panels show longer exposures. Arrows: endogenous YIPFα1A, arrowheads: exogenous HA‐tagged YIPFα1A. (B) Quantitation of endogenous (endo) versus exogenous (exo) YIPFα1A from anti‐YIPFα1A blots in (A, right). (C) Quantitation of WT versus Op YIPFα1A from anti‐HA blots in (A, left). (D) Northern blotting of total RNA (1 μg per lane) using a DIG‐labeled HA probe. Light (left) and heavy (right) exposures of the same blot are shown. Arrow, exogenous YIPFα1A mRNA. (E) Quantitation of total YIPFα1A mRNA from (D). (F) Northern blotting of total RNA (1 μg per lane) using a DIG‐labeled probe spanning the YIPFα1A coding sequence (CDS). Light (left) and heavy (right) exposures are shown. Arrowheads, endogenous isoforms; arrow, exogenous YIPFα1A mRNA. Representative image from three independent experiments. (G) Exogenous YIPFα1A mRNA relative to the sum of endogenous isoforms (larger and smaller) from (F). Statistics: Student's *t*‐test (**P < 0.01, ***P < 0.001).

Exogenous YIPFα1A protein expressed from WT CDS was barely detectable by anti‐HA immunoblotting, even with extended exposure times (Fig. 2A, left lower panel; arrowhead on the right), and remained below the detection threshold of the anti‐YIPFα1A antibody (Fig. 2A right lower panel; arrowhead on the right). These observations demonstrate that exogenous YIPFα1A protein expression from the WT CDS is extremely inefficient relative to endogenous YIPFα1A protein expression. In contrast, codon optimization markedly improved the exogenous YIPFα1A protein expression (Fig. 2A; Op), allowing robust detection by anti‐YIPFα1A antibody, which was unable to detect protein from WT CDS (Fig. 2A, right panels; Op).

Quantitative analysis revealed that the exogenous YIPFα1A protein produced from Op CDS was approximately 4‐fold of the endogenous protein (Fig. 2B) and approximately 30‐fold of the exogenous protein produced from the WT CDS (Fig. 2C). From these results, the amount of exogenous YIPFα1A protein expressed from the WT CDS is estimated to be roughly one‐eighth of the endogenous YIPFα1A protein level. Collectively, these findings indicate that the poor expression of exogenous YIPFα1A is primarily attributable to the enrichment of rare codons in its CDS.

### YIPFα1A expression is increased at the mRNA level by codon optimization

Rare‐codon enrichment is generally thought to reduce protein synthesis by decreasing translational efficiency. More recently, however, rare‐codon enrichment has also been shown to induce translation‐mediated mRNA decay, thereby reducing protein abundance at the mRNA level [36, 37]. To examine whether rare‐codon enrichment in the YIPFα1A CDS affect mRNA stability, northern blotting analysis was performed using an RNA probe targeting either the HA tag (Fig. 2D) or the YIPFα1A CDS (Fig. 2F). As expected, both the WT and the Op constructs produced a single major band of the anticipated size (approximately 1.1 kb) with either probe (Fig. 2D,F, arrows). However, mRNA levels were dramatically higher in the Op mutant compared with the WT (Fig. 2D), showing approximately 30‐fold increase (Fig. 2E), comparable to the magnitude observed for protein expression (Fig. 2C). These findings indicate that the poor exogenous expression of YIPFα1A is largely attributable to reduced mRNA abundance.

Two major endogenous YIPFα1A mRNAs (2.0 kb and 3.2 kb) were detected by the YIPFα1A CDS probe in nontransfected HEK293 cells (Fig. 2F; Heavy, N, arrowheads), consistent with our earlier finding that most human tissues express two similarly sized isoforms [27]. The exogenously expressed WT YIPFα1A mRNA level was higher than the endogenous mRNA level (Fig. 2F; N vs. WT). Quantitative analysis comparing the exogenous WT mRNA to the combined amount of the two endogenous mRNA isoforms revealed that exogenous expression was approximately 20‐fold higher (Fig. 2G). This result contrasts sharply with the extremely low level of exogenous protein expression from the WT YIPFα1A CDS relative to endogenous protein expression (Fig. 2A–C), suggesting that protein expression from the endogenous mRNA is far more efficient than that from exogenous WT mRNA.

### Regulation of endogenous YIPFα1A expression at the mRNA level

**Fig. 3:**
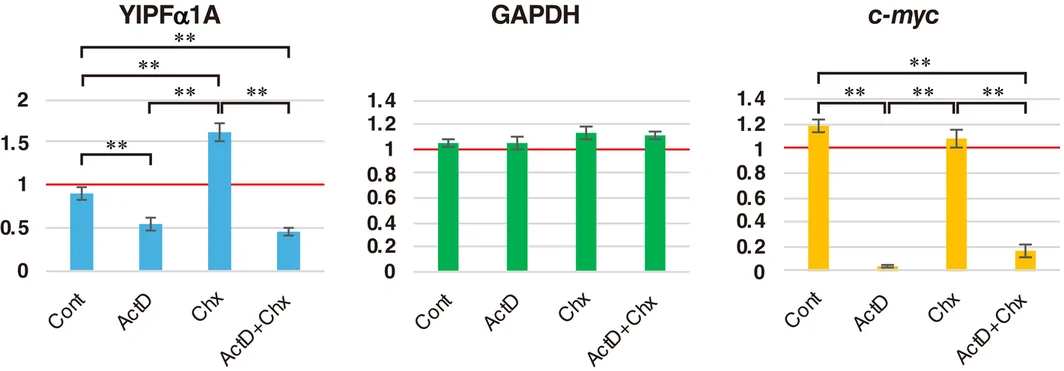
Changes in mRNA levels after inhibition of transcription and/or translation. HEK293 cells were treated for 6 h with actinomycin D (ActD), cycloheximide (Chx), both (ActD+Chx), or vehicle (DMSO, C) for 6 h. Total RNA was isolated and analyzed by qPCR with gene‐specific primers targeting coding sequence (CDS), as described in Methods. mRNA levels were expressed as ratios relative to the untreated control cells. Data are presented as mean with error bars indicating the standard error of the mean (SEM); *n* = 3. Statistics: One‐way ANOVA with Fisher's LSD (**P < 0.01, no mark: not significant).

The results described above strongly suggest that endogenous YIPFα1A expression is regulated at the mRNA level. To examine this possibility, we assessed the stability of endogenous YIPFα1A mRNA following transcriptional inhibition. HEK293 cells were treated with actinomycin D (1 μg·mL^−1^) for 6 h, total RNA was extracted, and endogenous mRNA levels were quantified by qPCR using primer sets targeting the CDSs (Fig. 3). As expected, the mRNA level of glyceraldehyde 3‐phosphate dehydrogenase (GAPDH), a commonly used housekeeping control with a long half‐life [38, 39], remained unchanged. In contrast, the mRNA level of *c‐myc*, a transcript known to have a short half‐life [40, 41], was markedly reduced (~ 5% of the control) following actinomycin D treatment. Endogenous YIPFα1A mRNA levels decreased to ~ 50% of control, indicating that the half‐life of YIPFα1A mRNA is substantially shorter than that of GAPDH. These observations support the idea that rare‐codon enrichment in the YIPFα1A CDS contributes to reduced endogenous mRNA abundance.

Because the reduction in YIPFα1A mRNA levels may result from translation‐dependent effects on mRNA stability [36, 37], we next examined whether inhibiting translation influences YIPFα1A mRNA abundance. Cycloheximide, an inhibitor commonly used to assess nonsense‐mediated decay [42, 43], was applied to HEK293 cells (100 μg·mL^−1^) for 6 h. GAPDH mRNA levels remained unchanged when treated with cycloheximide alone, or in combination with actinomycin D, confirming its validity as a control.

*c‐myc* mRNA levels were unaffected by cycloheximide treatment alone, whereas they were strongly reduced to ~ 15% of control under simultaneous cycloheximide and actinomycin D treatment. This result was somewhat unexpected, because cycloheximide has been reported to stabilize *c‐myc* mRNA in other cultured cells (CHO and Swiss 3T3) [41]. Under transcriptional inhibition, cycloheximide caused a slight increase in *c‐myc* mRNA compared with actinomycin D treatment alone (reduced to ~ 4%), although the effect was only marginally significant (*P* < 0.05; one‐way Student's *t*‐test). These observations suggest that, in HEK293 cells, pathways other than translation‐dependent decay constitute the major route for *c‐myc* mRNA clearance.

Interestingly, YIPFα1A mRNA levels increased significantly (~ 1.5 fold) upon cycloheximide treatment. However, simultaneous cycloheximide and actinomycin D treatment reduced YIPFα1A mRNA to the same level observed with actinomycin D alone. These results indicate that cycloheximide increases YIPFα1A mRNA levels only when transcription is active, and not by stabilizing pre‐existing transcripts. Therefore, the cycloheximide‐induced increase in YIPFα1A mRNA most likely reflects transcriptional activation rather than enhanced mRNA stability resulting from translation inhibition.

### 3′ UTR enhances YIPFα1A expression

Because endogenous YIPFα1A mRNA yields much higher protein expression than exogenously expressed YIPFα1A mRNA lacking the 3′ UTR (Fig. 2), we hypothesized that the extended 3′ UTR—another shared feature of poorly expressed *α*‐subunits—positively contributes to YIPFα1A expression. The 3′ UTR is known to regulate protein expression by modulating mRNA stability and/or intracellular localization [32, 33, 34, 44]. Therefore, we sought to determine the functional significance of the extended 3′ UTR of YIPFα1A.

As described above, two isoforms of endogenous YIPFα1A mRNA were detected in HEK293 cells (Fig. 2F, Heavy, N). The sizes of the larger (3.2 kilobase [kb]) and smaller (2.0 kb) isoforms correspond closely to the full‐length sequence (3395 nucleotide[nt]s) and the truncated sequence ending at the annotated internal poly(A) site (2284 nts) of the YIPFα1A reference cDNA sequence (NM_001024947), respectively.

**Fig. 4:**
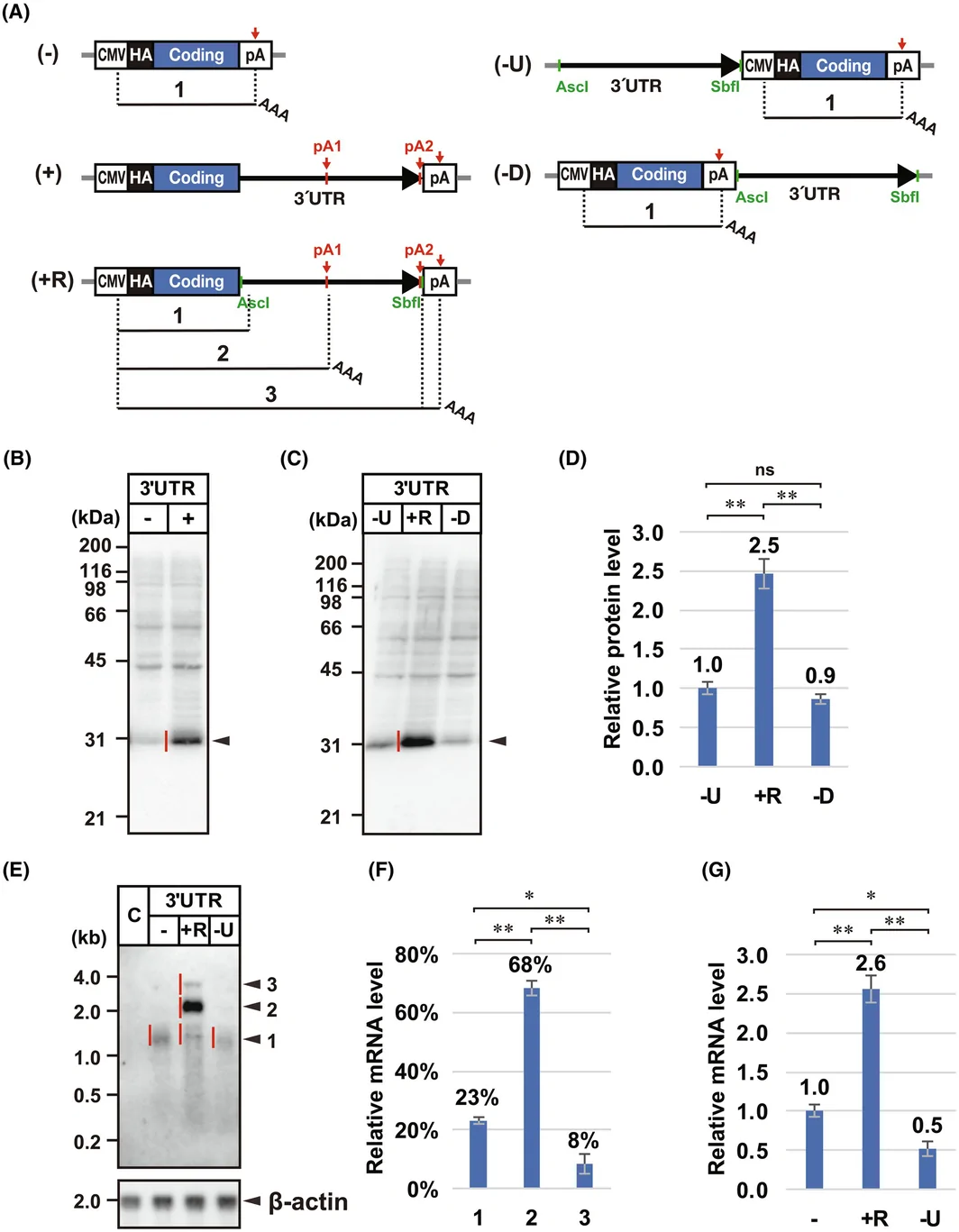
The YIPFα1A 3′ untranslated region (UTR) increases expression. HEK293 cells were transfected with YIPFα1A constructs harboring different 3′ UTR configurations and analyzed by western and northern blotting. Molecular weight/size markers are shown on the left. Red lines on the left of each lane indicate quantitation regions. Data are presented as mean with error bars indicating the standard error of the mean (SEM); *n* = 3. (A) Schematic of constructs. (−): coding sequence (CDS) only; (+): native 3′ UTR directly appended to the CDS; (+R), 3′ UTR with introduced restriction sites (AscI, SbfI, green); (−U), 3′ UTR inserted upstream of the CMV promoter; (−D), 3′ UTR inserted downstream of the SV40 poly(A) signal. Red arrows indicate poly(A) signals (pA1 and pA2) within the YIPFα1A 3′ UTR, and the SV40 poly(A). Predicted transcripts corresponding to Bands 1–3 are indicated below. (B, C) Representative western blotting from three independent experiments; arrowheads, YIPFα1A protein. (D) YIPFα1A protein levels relative to control (−U). (E) Representative northern blotting using DIG‐labeled probe spanning the YIPFα1A CDS showing three major bands (1, 2, and 3, arrowheads). A *β*‐actin control blot (probe spanning the *β*‐actin CDS) from the same samples is shown below. Red lines on the left of each lane indicate regions used for quantitation. (F) Relative abundance (%) of Bands 1, 2, and 3. (G) Total mRNA from 3′ UTR‐containing construct (+R) relative to control (−). For (+R), Bands 2 and 3 were summed. Statistics: One‐way ANOVA with Fisher's LSD. (*P < 0.05, **P < 0.01, no mark: not significant).

Having confirmed that YIPFα1A mRNA containing the extended 3′ UTR is expressed endogenously, we next examined its functional role. For this purpose, we constructed an expression plasmid encoding YIPFα1A mRNA with its native, extended 3′ UTR (Fig. 4A, +). Constructs with (+) or without (−) the 3′ UTR were transfected into HEK293 cells, and YIPFα1A protein expression was compared by western blotting (Fig. 4B). Strikingly, inclusion of the 3′ UTR enhanced YIPFα1A protein expression (+ vs −).

To control for the possibility that insertion of the 3′ UTR affected transcription from the plasmid or that differences in plasmid size influenced transfection efficiency, two additional control constructs were generated. In these constructs, the 3′ UTR was inserted either upstream of the CMV promoter (Fig. 4A, −U) or downstream of the poly(A) signal (−D). In addition, restriction sites (AscI, SbfI) were introduced flanking the 3′ UTR to facilitate plasmid construction. As shown in Fig. 4C,D, addition of the 3′ UTR (+R) significantly enhanced protein expression—approximately 2.5‐fold—compared with both control constructs (−U, −D). These results strongly indicate that the YIPFα1A 3′ UTR plays an active role in enhancing protein expression.

### 3′ UTR increases the expression of YIPFα1A mRNA

Northern blotting was next performed to analyze the size (Fig. 4E) and abundance (Fig. 4F,G) of the exogenously expressed YIPFα1A mRNA derived from the construct containing the 3′ UTR (+R). A single major band was detected at approximately 1.1 kb (Fig. 4E, Band 1) for the control constructs lacking the 3′ UTR (−, −U), consistent with the predicted transcript size from the control constructs (1052 nts). In contrast, three distinct bands were observed for the construct containing the 3′ UTR (Fig. 4E, +R; Bands 1, 2, and 3). The estimated sizes of Bands 1, 2, and 3 were 1.1 kilobase (kb), 2.3 kb, and 3.4 kb, respectively.

Band 3 likely represents the fully transcribed mRNA terminated at the poly(A) signal at the 3′ end of the 3′ UTR (Fig. 4A; pA2), although we cannot exclude the possibility that some signal originates from transcripts extending to the downstream SV40 poly(A) signal (Fig. 4A; red arrow to the right of pA2). Band 2 was the most abundant species (Fig. 4F) and is predicted to correspond to transcripts terminated at the internal poly(A) site (Fig. 4A; pA1). Band 1, although present in the +R construct, was unexpected, because no poly(A) signal is predicted to generate a transcript of that size. This band is most likely a partial degradation product, a possibility discussed further below.

Importantly, overall mRNA abundance was markedly increased in the presence of the 3′ UTR (Fig. 4G). These results strongly suggest that the 3′ UTR enhances YIPFα1A protein expression in part by increasing YIPFα1A mRNA levels.

### Functional analysis of larger and smaller mRNA isoforms

**Fig. 5:**
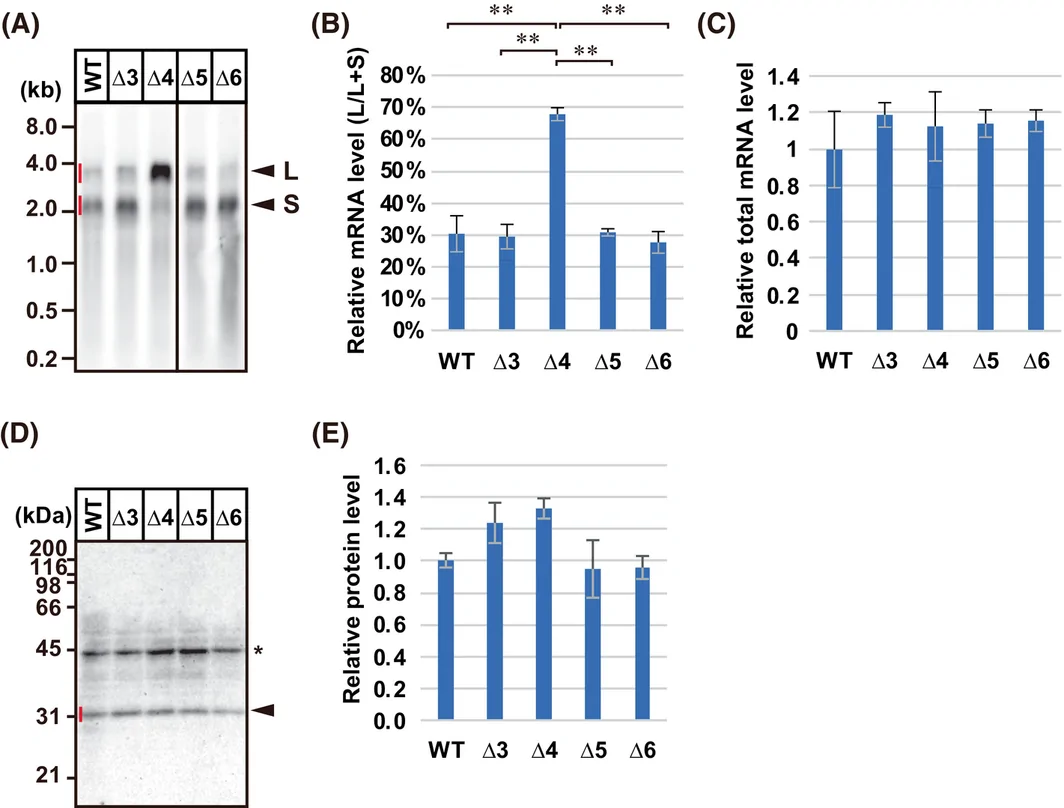
Functional role of the internal poly(A) signal in the YIPFα1A 3′ untranslated region (UTR). HEK293 cells were transfected with constructs harboring mutations in candidate internal poly(A) signals (Δ3–Δ6) within the 3′ UTR and analyzed by northern and western blotting. Markers are shown on the left; red lines on the left of each lane indicate regions used for quantitation. Data are presented as mean with error bars indicating the standard error of the mean (SEM); *n* = 3. (A) Representative northern blotting [DIG‐labeled probe spanning the YIPFα1A coding sequence (CDS)] showing larger (L) and smaller (S) mRNA isoforms (arrowheads). (B) Percentage of the larger isoform for each construct. (C) Total YIPFα1A mRNA (L + S) relative to WT. (D) Representative western blotting; arrowhead, YIPFα1A; asterisk, nonspecific band. (E) YIPFα1A protein levels relative to WT. Statistics: One‐way ANOVA with Fisher's LSD (**P < 0.01; no mark: not significant).

As described above, northern blotting analyses revealed that exogenous expression of YIPFα1A containing its 3′ UTR produced larger and smaller mRNA isoforms whose sizes matched well with those of endogenous YIPFα1A transcripts. We, therefore, examined the functional significance of these two isoforms. As a first step, the poly(A) signal responsible for generating the smaller isoform was examined by introducing mutations into candidate poly(A) signal sequences located near the center of the 3′ UTR, where appropriately sized transcripts were expected. Four candidate sequences were selected, and the last adenine was mutated to guanine—a change known to abolish poly(A) signal activity (Table 3, ∆3 ~ 6) [45]. Among these mutants, only the ∆4 mutant exhibited a clear increase in the larger isoform accompanied by a decrease in the smaller isoform (Fig. 5A,B). This finding indicates that the sequence at positions 1104–1110 (AUUAAA)—the annotated internal poly(A) signal—is indeed responsible for producing the smaller isoform.

**Table 3:** Mutation of predicted poly A sites.

Mutant name	Base No.^a^	WT	Mutant
∆3	1010–1016	AAUAAA	AAUAAG
∆4	1104–1110	AUUAAA	AUUAAG
∆5	1260–1266	AUUAAA	AUUAAG
∆6	1296–1302	AUUAAA	AUUAAG

^a^ Counted from the 5′ end of the 3′ UTR.

**Table 4:** Statistical significance^a^.

M	1–1118	1116–2230	1–746	374–1118	1–373	374–746	747–1118	1–200	GAPDH
1–2230	NS	*P* < 0.05	NS	*P* < 0.01	NS	*P* < 0.01	*P* < 0.01	*P* < 0.01	*P* < 0.05
1–1118	‐	NS	NS	*P* < 0.05	NS	NS	*P* < 0.01	*P* < 0.01	NS
1116–2230	‐	‐	NS	NS	*P* < 0.05	NS	*P* < 0.05	*P* < 0.01	NS
1–746	‐	‐	‐	P < 0.01	NS	P < 0.05	P < 0.01	P < 0.01	NS
374–1118	‐	‐	‐	‐	*P* < 0.01	NS	NS	*P* < 0.01	NS
1–373	‐	‐	‐	‐	‐	*P* < 0.01	*P* < 0.01	*P* < 0.01	NS
374–746	‐	‐	‐	‐	‐	‐	NS	*P* < 0.01	NS
747–1118	‐	‐	‐	‐	‐	‐	‐	*P* < 0.01	*P* < 0.05
1–200	‐	‐	‐	‐	‐	‐	‐	‐	*P* < 0.01

S	1–1118	1116–2230	1–746	374–1118	1–373	374–746	747–1118	1–200	GAPDH
1–2230	NS	NS	NS	NS	NS	NS	NS	*P* < 0.01	*P* < 0.05
1–1118	‐	*P* < 0.01	NS	*P* < 0.05	*P* < 0.01	*P* < 0.01	*P* < 0.05	*P* < 0.01	*P* < 0.01
1116–2230	‐	‐	*P* < 0.05	NS	NS	NS	NS	*P* < 0.01	NS
1–746	‐	‐	‐	NS	*P* < 0.05	*P* < 0.05	NS	*P* < 0.01	*P* < 0.05
374–1118	‐	‐	‐	‐	NS	NS	NS	*P* < 0.01	NS
1–373	‐	‐	‐	‐	‐	NS	NS	*P* < 0.01	NS
374–746	‐	‐	‐	‐	‐	‐	NS	*P* < 0.01	NS
747–1118	‐	‐	‐	‐	‐	‐	‐	*P* < 0.01	NS
1–200	‐	‐	‐	‐	‐	‐	‐	‐	*P* < 0.01

D	1–1118	1116–2230	1–746	374–1118	1–373	374–746	747–1118	1–200	GAPDH
1–2230	NS	*P* < 0.01	NS	*P* < 0.01	*P* < 0.05	*P* < 0.01	*P* < 0.01	NS	*P* < 0.01
1–1118	‐	*P* < 0.01	NS	*P* < 0.01	NS	*P* < 0.01	*P* < 0.01	NS	*P* < 0.01
1116–2230	‐	‐	*P* < 0.01	NS	*P* < 0.05	NS	*P* < 0.05	*P* < 0.01	NS
1–746	‐	‐	‐	*P* < 0.01	NS	*P* < 0.01	*P* < 0.01	NS	*P* < 0.01
374–1118	‐	‐	‐	‐	*P* < 0.01	NS	NS	*P* < 0.01	NS
1–373	‐	‐	‐	‐	‐	*P* < 0.01	*P* < 0.01	NS	*P* < 0.05
374–746	‐	‐	‐	‐	‐	‐	NS	*P* < 0.01	NS
747–1118	‐	‐	‐	‐	‐	‐	‐	*P* < 0.01	NS
1–200	‐	‐	‐	‐	‐	‐	‐	‐	*P* < 0.01

^a^ Statistical significance was determined using one‐way ANOVA followed by Fisher's LSD test.

Notably, quantitation of total mRNA (i.e., the combined abundance of larger and smaller isoforms) showed that disruption of the internal poly(A) signal in the ∆4 mutant did not affect the overall mRNA level (Fig. 5C). Consistent with this observation, YIPFα1A protein expression was unchanged by the mutation (Fig. 5D,E). These results suggest that the smaller isoform, which contains only the proximal region of the 3′ UTR, carries an element required for enhancing YIPFα1A expression.

### Functional mapping of 3′ UTR regulatory elements

**Fig. 6:**
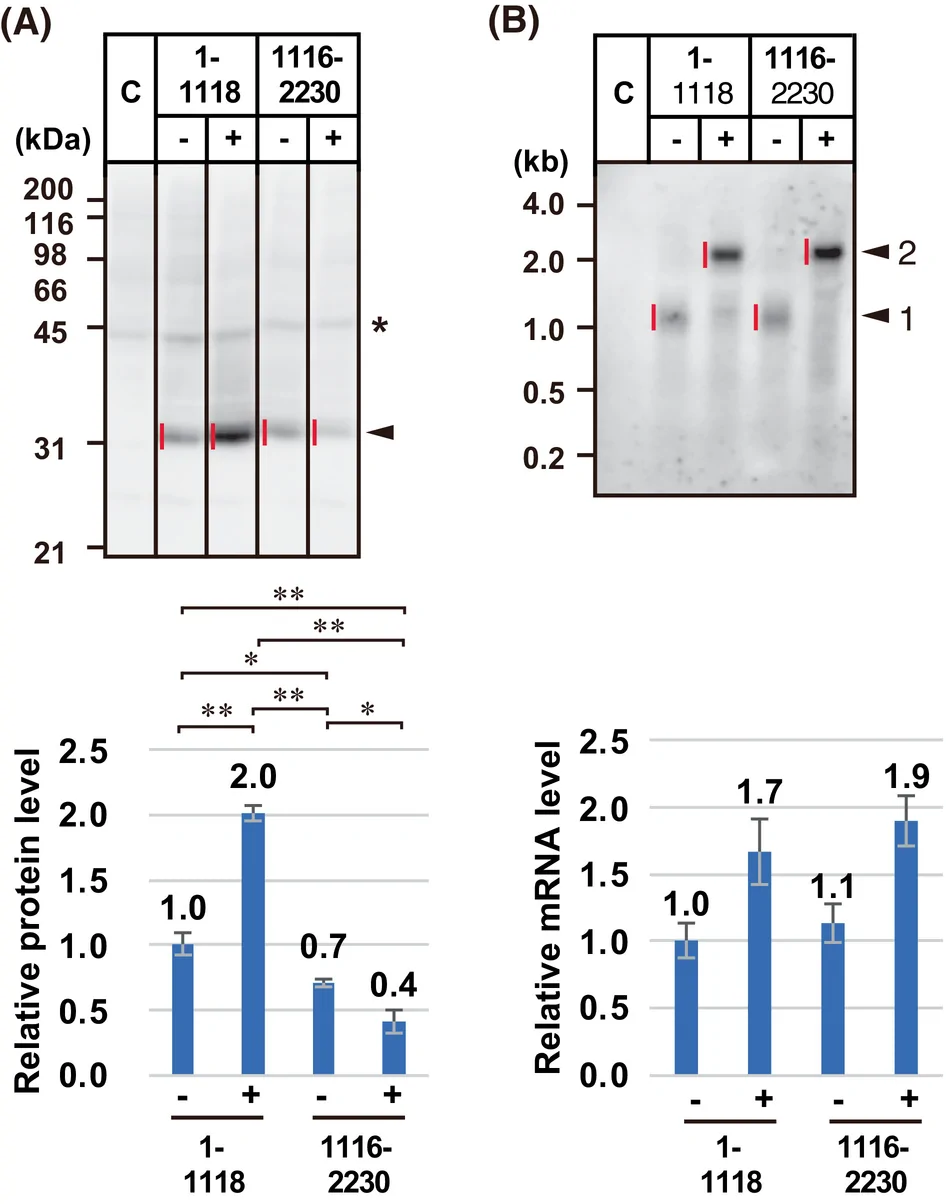
Functional analysis of proximal and distal regions of the YIPFα1A 3′ untranslated region (UTR). Constructs harboring the YIPFα1A coding sequence (CDS) with either the proximal (1–1118) or distal (1116–2230) half of the 3′ UTR were tested, with fragments placed either at the 3′ end of the CDS or at the control position (upstream of the CMV promoter; −U in Fig. 4A). Markers are shown on the left; red lines on the left of each lane indicate regions used for quantitation. Data are presented as mean with error bars indicating the standard error of the mean (SEM); *n* = 3. (A) Western blotting (upper) and quantitation (lower). Arrowhead, YIPFα1A; asterisk; nonspecific band. (B) Northern blotting (DIG‐labeled YIPFα1A CDS probe; upper) and quantitation (lower). Arrowhead 1: CDS only transcript; arrowhead 2: CDS + 3′ UTR fragment transcript. Statistics: one‐way ANOVA with Fisher's LSD (*P < 0.05, **P < 0.01; no mark: not significant).

To investigate the functional contribution of the proximal region of the 3′ UTR, the full 3′ UTR sequence was divided into proximal (1–1118) and distal (1116–2230) fragments at the internal poly(A) signal responsible for generating the smaller isoform (Fig. 4A; pA1). These fragments were inserted either downstream of the CDS or into a control position to construct expression plasmids, and their effects were analyzed as described above. Western blotting analysis showed that the proximal fragment (1–1118), but not the distal fragment (1116–2230), increased YIPFα1A protein expression (Fig. 6A). This result supports the hypothesis that the proximal region of the 3′ UTR contains an element required for enhancing YIPFα1A protein expression.

Northern blotting revealed that mRNA levels increased upon addition of the proximal fragment, reaching levels comparable to those observed at the protein level (Fig. 6B). However, unexpectedly, addition of the distal fragment also increased mRNA levels to a degree comparable to that produced by the proximal fragment. This was surprising because experiments using the full‐length 3′ UTR indicated that the increase in protein expression was primarily attributable to elevated mRNA abundance (Fig. 4E,G). These findings suggest the presence of an additional regulatory mechanism acting at the post‐transcriptional level: either the proximal region enhances translation, or the distal region suppresses translation efficiency. These possibilities were further examined through a deletion analysis, as described below.

### Identification of a 3′ UTR region that enhances protein expression

**Fig. 7:**
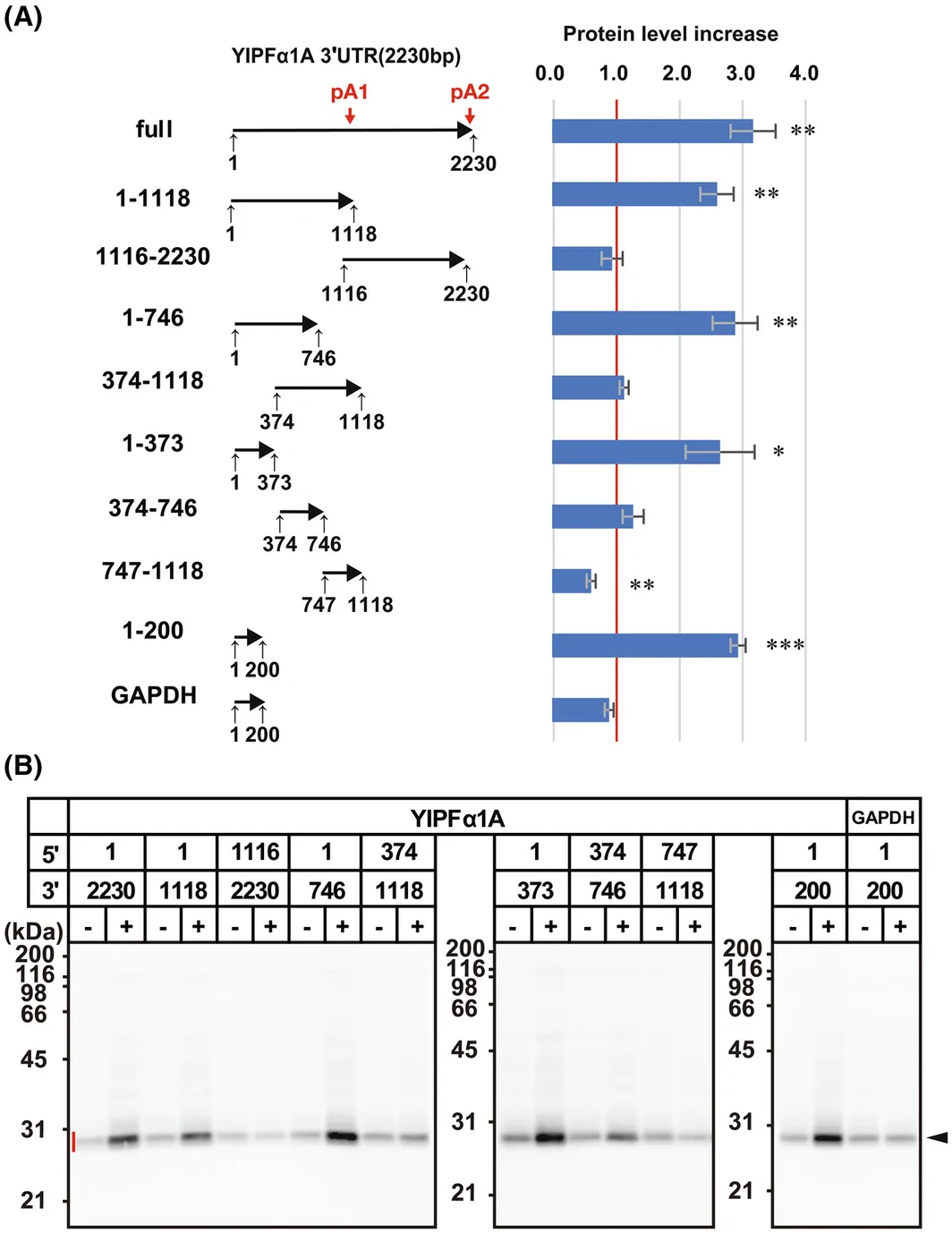
Identification of 3′ untranslated region (UTR) regions that affect protein expression. HEK293 cells were transfected with YIPFα1A coding sequence (CDS) constructs (HiBiT‐tagged) carrying various YIPFα1A or GAPDH 3′ UTR fragments. Fragments were positioned either at the 3′ end of the CDS (+) or at the control position (−; downstream of the SV40 poly(A) signal; −D in Fig. 4A). Markers are shown on the left; red lines on the left of each lane indicate regions used for quantitation. Data are presented as mean with error bars indicating the standard error of the mean (SEM); *n* = 3. (A) Quantitation and schematic summary. Left; schematic of deletion constructs with poly(A) positions (pA1, pA2; red arrows) and fragment boundaries. Right; fold increase in protein levels, computed as (+)/(−) where (+) denotes the 3′ UTR‐fragment construct and (−) the corresponding control; red line indicates no change (ratio = 1). Statistics: Student's *t*‐test (*P < 0.05, **P < 0.01, ***P < 0.001, no mark: not significant). (B) Representative Nano‐Glo chemiluminescence image; arrowhead, YIPFα1A. Fragment boundaries (5′/3′ nt positions) are indicated above.

Various deletion fragments of the 3′ UTR (Fig. 7A, left) were generated and inserted either downstream of the CDS (+) or into the control position (−; downstream of the SV40 poly(A) signal, Fig. 4A, ‐D) to create expression plasmids. These constructs were analyzed by western blotting as described above (Fig. 7B). For efficient detection, the N‐terminal HA‐tag was replaced with a HiBiT‐tag. Notably, intracellular localization of the protein was unchanged by this tag replacement (data not shown). Quantitation was performed as previously described, and results were presented as fold increases in protein expression, calculated as the ratio of protein produced from the construct containing the 3′ UTR fragment (+) to that produced from the corresponding control (−) (Fig. 7A).

All fragments containing the region of the 3′ UTR immediately downstream of the CDS (1–373) increased YIPFα1A protein expression to similar levels (full, 1–1118, 1–746, 1–373). In contrast, all fragments lacking this region (1116–2230, 374–1118, 374–746, 747–1118) failed to enhance expression. These findings clearly demonstrate that the proximal region of the 3′ UTR adjacent to the CDS (1–373) is necessary and sufficient to promote YIPFα1A protein expression. Moreover, the shortest fragment examined (1–200) also increased expression, whereas a size‐matched 3′ UTR fragment from the glyceraldehyde‐3‐phosphate dehydrogenase (GAPDH) did not. These observations strongly suggest that a regulatory element responsible for enhancing YIPFα1A protein expression resides within the proximal 200 nts of the 3′ UTR.

### Identification of a 3′ UTR region that enhances mRNA expression

**Fig. 8:**
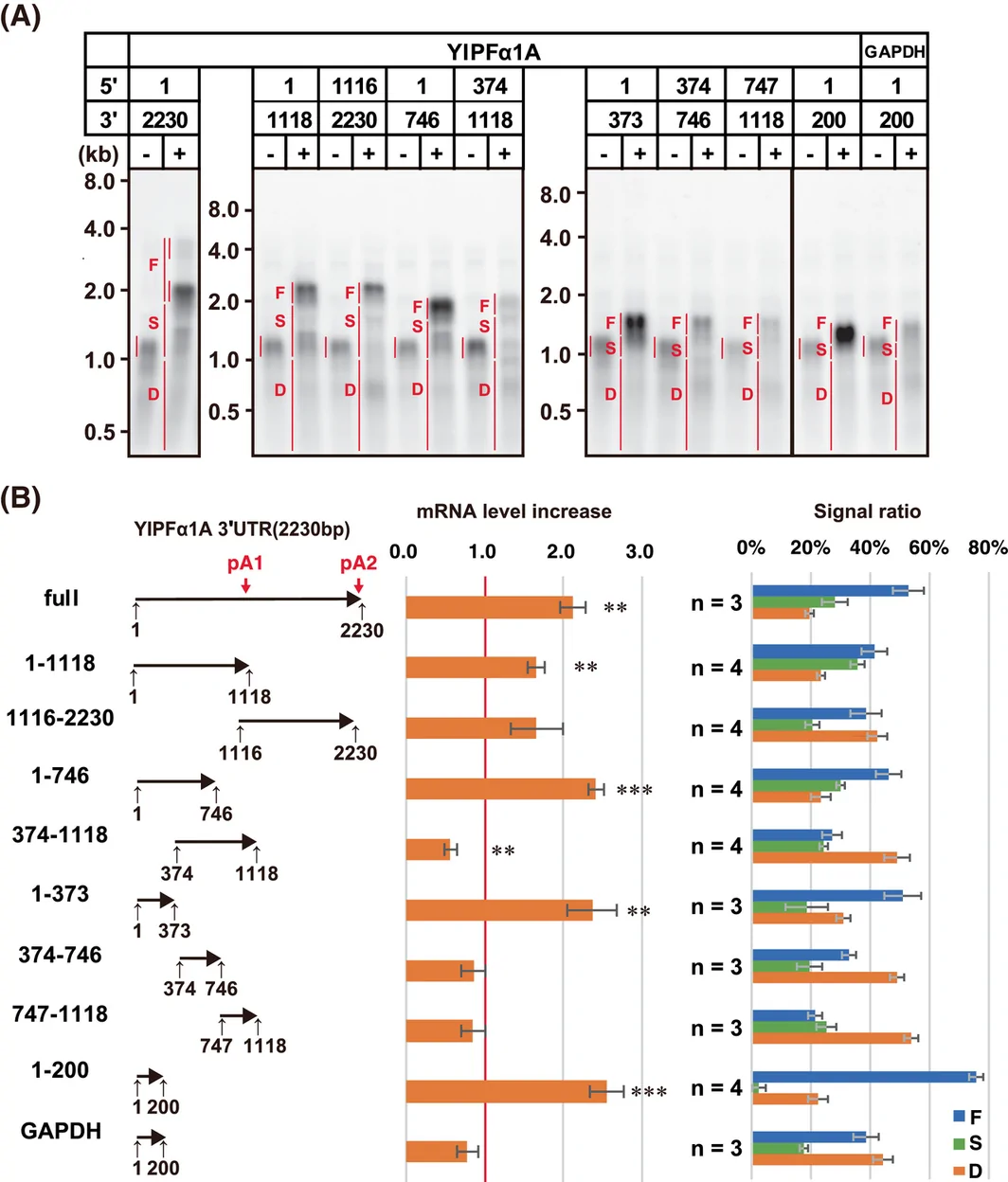
Identification of a 3′ untranslated region (UTR) region that affects mRNA expression. Northern blotting of constructs described in Fig. 6. Markers are shown on the left, red lines labeled F (full‐length), S (smaller than full‐length), and D (degraded) on the left of each lane indicate regions used for quantitation. Data are presented as mean with error bars indicating the standard error of the mean (SEM); *n* = 3 or 4 (indicated on the left of right bar graph). (A) Representative northern blot using a DIG‐labeled probe spanning the YIPFα1A coding sequence (CDS). (B) Quantitation and analysis. Left; schematic (as in Fig. 7A). Middle; fold increase in mRNA (+)/(−); red line indicates no change (ratio = 1). Right; relative abundance F, S, and D species per each construct. Statistics: Student's *t*‐test (**P < 0.01, ***P < 0.001, no mark: not significant). Statistical comparisons of signal ratios between constructs are summarized in Table 4.

Next, mRNA expression was analyzed by northern blotting. As shown in Fig. 8A, mRNAs of the expected sizes were detected for all constructs containing 3′ UTR fragments and their corresponding controls, indicating that the introduced 3′ UTR sequences were successfully transcribed along with the CDS. We then compared the abundance of the full‐length transcript (Fig. 8A; F) following the same procedure used in Fig. 5B (Fig. 8B, center bar graph; F). For constructs containing the full‐length 3′ UTR fragment (1–2230), the two larger and smaller isoforms were detected. These were quantitated separately and then summed. Results were expressed as fold increases in mRNA abundance, calculated as the ratio of full‐length transcript from constructs containing a 3′ UTR fragment (+) to that from the corresponding control (−).

All fragments containing the region of the 3′ UTR adjacent to the CDS (1–373) increased YIPFα1A mRNA expression to similar levels (~ 2‐fold; full, 1–1118, 1–746, 1–373). In contrast, fragments lacking this region (374–1118, 374–746, 747–1118) did not increase mRNA abundance, except for the distal 3′ UTR fragment (1116–2230). As observed for protein expression, the shortest fragment (1–200) also increased mRNA levels (~ 2.5 fold), whereas the size‐matched GAPDH 3′ UTR fragment showed no such effect. Together, these results strongly suggest that the proximal region of the 3′ UTR (1–200) promotes YIPFα1A protein expression by increasing mRNA abundance.

### The distal region of the 3′ UTR increases mRNA expression but suppresses translation

Curiously, the distal fragment of the 3′ UTR (1116–2230) increased mRNA levels but did not enhance protein expression, consistent with earlier observations (Fig. 6). Given the clear function of the proximal 3′ UTR region (1–200) in producing correlated increases in both mRNA and protein levels, these results suggest that the distal region suppresses translation, rather than the proximal region enhances translation.

**Fig. 9:**
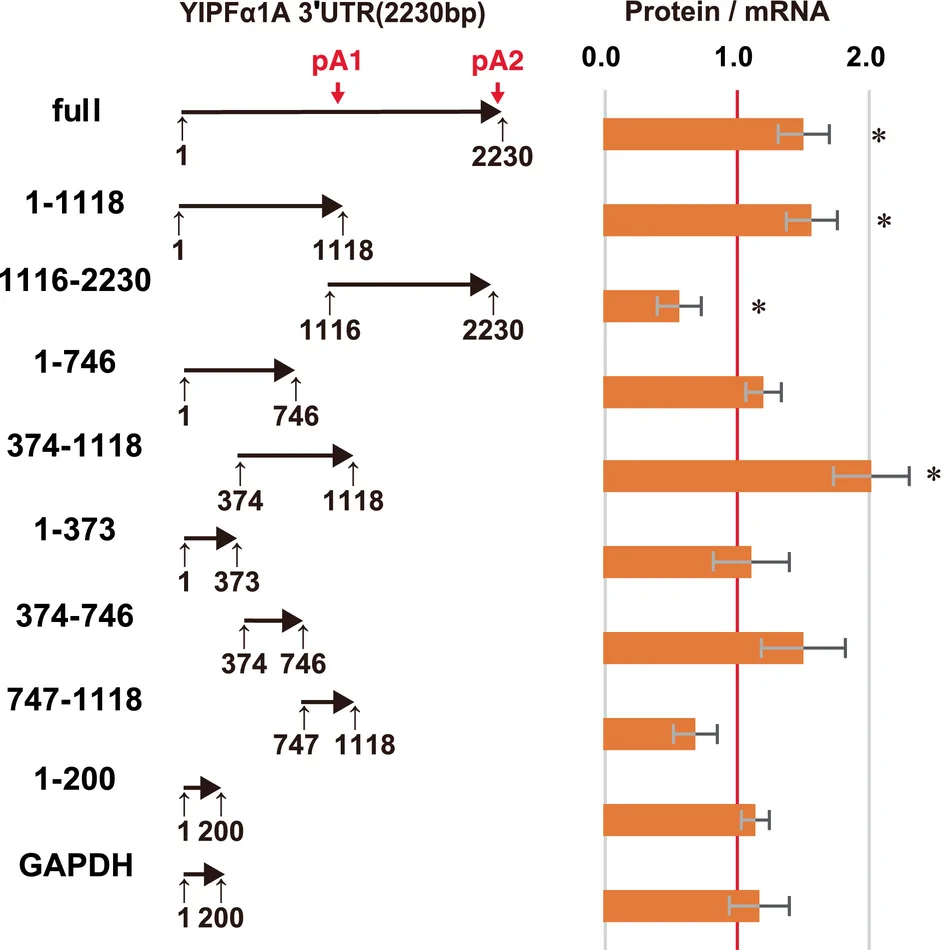
Protein‐to‐mRNA expression ratio analysis. Left; schematic of 3′ UTR deletions (as in Figs 7, 8). Right; protein abundance relative to mRNA abundance was computed using data from Figs 7 and 8 (*fold* [protein]/*fold*[mRNA]). The standard error of the mean (SEM) was calculated using the standard error propagation formula. Because the sample sizes (*n*) of the data differed, the calculation was conducted under the assumption of the smallest sample size (*n* = 3). Data are presented as mean with error bars indicating the SEM, red line indicates no change (ratio = 1). Statistics: Student's *t*‐test (*P < 0.05, no mark: not significant).

To examine how the 3′ UTR regulates protein expression independently of changes in mRNA abundance, we compared fold‐changes in protein levels with corresponding fold‐changes in mRNA levels by calculating the protein‐to‐mRNA ratio (Fig. 9). As expected, the distal fragment (1116–2230) exhibited a markedly reduced protein‐to‐mRNA ratio (~ 0.70) compared with the full‐length 3′ UTR and the proximal fragments (1–1118), which showed ratios > 1.0. These results indicate that protein output per mRNA is substantially lower (~ 60%) for transcripts containing the distal fragment than for those containing proximal fragments. The basis of this suppression is addressed in the Discussion.

Interestingly, the fragment spanning nts 374–1118 showed an apparently high protein/mRNA ratio; this was attributable to reduced mRNA levels caused by increased degradation (discussed below); therefore, the biological significance of this observation remains uncertain.

In addition, northern blotting analysis revealed prominent signals corresponding to smaller transcripts across all constructs (Fig. 8A; S and D). As described earlier for the full‐length 3′ UTR construct (Fig. 4E), these species are most likely degradation products of the fully transcribed mRNA (Fig. 8A; F). The sizes and relative intensities of these smaller products varied among constructs (Fig. 8A). Notably, substantial signals were detected for transcripts equal to or larger than the control's full‐length transcript (Fig. 8A, S). To quantitatively assess these differences, each lane was divided into three regions as we mentioned above: (1) the full‐length transcript (Fig. 8A, F); (2) transcripts smaller than the full‐length but equal to or larger than the control's full‐length transcript (S); and (3) transcripts smaller than the full‐length transcript of the control (D). Species S and D were likely degradation intermediates. Quantitation was performed as described in the Methods, and the percentage of signal in each region was determined for every sample (Fig. 8B, right).

Notably, the fraction of degradation products smaller than the CDS (D) was substantially higher for constructs lacking the 1–200 region (1116–2230, 374–1118, 374–746, 747–1118) and for the GAPDH 3′ UTR control, whereas it was lower for constructs containing the 1–200 region of the YIPFα1A 3′ UTR. These findings strongly suggest that nts 1–200 of the YIPFα1A 3′ UTR contribute to mRNA stabilization.

**Fig. 10:**
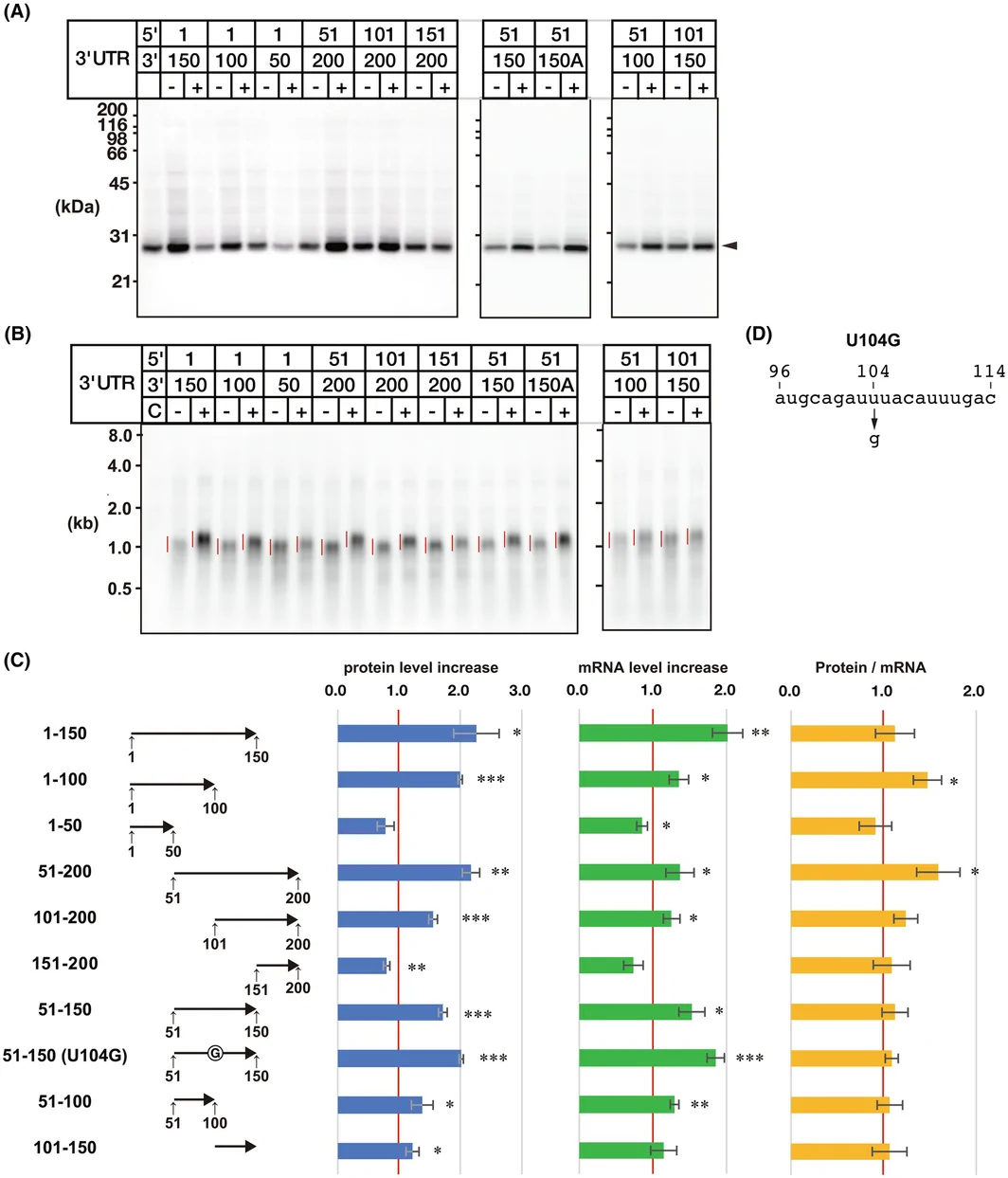
Identification of minimal mRNA stabilizing element. 3′ untranslated region (UTR) deletion mutants were constructed and analyzed as in Figs 7, 8, 9. (A) Representative Nano‐Glo chemiluminescence image; arrowhead, YIPFα1A. (B) Representative northern blotting (DIG‐labeled probe spanning the YIPFα1A coding sequence (CDS)). (C) Quantitation and analysis. Left; schematic of mutants. Middle left; fold increase in protein expression. Middle right; fold increase in mRNA expression. Right; protein‐to‐mRNA ratio; red line indicates no change (ratio = 1). Data are presented as mean with error bars indicating the standard error of the mean (SEM); *n* = 3. Error values on right bar graph were derived with the standard error propagation formula. Statistics: Student's *t*‐test (*P < 0.05, no mark: not significant). (D) Uracil at the nt104 was mutated to guanine (U104G). Surrounding sequence is shown.

To further define the region responsible for this effect, we performed a more detailed deletion analysis. Various 3′ UTR deletion mutants and their respective controls were constructed and analyzed as described previously (Fig. 10). Both protein and mRNA levels were increased to a similar extent (~ 2‐fold), comparable to the effect of the 1–200 fragment, only in mutants containing nts 51–150. Additional deletions from either the 5′ or 3′ side substantially reduced the increase in mRNA abundance. These observations clearly indicate that nts 51–150 of the YIPFα1A 3′ UTR constitute the minimal element required for mRNA stabilization.

Finally, we examined the AUUUA motif located near the center of the 51–150 region, which has been reported to mediate HuR binding (Fig. 10D) [46]. However, mutating the central uracil to guanine (U104G) within the 51–150 construct did not produce any significant effect.

## Discussion

### mRNA destabilization via rare‐codon enrichment in the CDS

Exogenous YIPFα1A protein expression was strongly repressed by the enrichment of rare codons in the CDS. This repression occurred primarily at the mRNA level, as codon optimization of CDS increased mRNA abundance to a level comparable to the corresponding protein expression (Fig. 2A,C–E; approximately 30‐fold). The suppression in mRNA expression is thought to result from enhanced mRNA degradation induced by rare‐codon enrichment, as reported previously [36, 47, 48, 49, 50, 51, 52, 53].

Inhibition of transcription with actinomycin D markedly reduced endogenous YIPFα1A mRNA, indicating a significantly shorter half‐life and corroborating that endogenous YIPFα1A expression is also controlled by mRNA stability (Fig. 3). To test whether translation influences endogenous YIPFα1A mRNA stability, we inhibited translation with cycloheximide, which is commonly used to assess nonsense‐mediated decay [42, 43]. However, unexpectedly, the results did not support a role for translation in stabilizing endogenous YIPFα1A mRNA. Therefore, the mechanisms regulating the stability of endogenous YIPFα1A mRNA remain to be elucidated. Because the level of exogenous YIPFα1A mRNA exceeded the endogenous level by more than 20‐fold (Fig. 2F,G), degradation driven by translation of the rare‐codon‐enriched CDS may have predominated under our exogenous expression conditions.

Although our experiments did not demonstrate translation‐induced decay of endogenous YIPFα1A mRNA, our results clearly indicate that endogenous YIPFα1A mRNA is actively degraded by specific cellular mechanisms. Determining whether, and to what extent, rare‐codon enrichment within the CDS contributes to the regulation of endogenous YIPFα1A mRNA expression will require further investigation.

### Induction of YIPFα1A transcription by translational inhibition

Notably, translational inhibition by cycloheximide markedly increased YIPFα1A mRNA in HEK293 cells (Fig. 3), likely via transcriptional activation. Although more detailed analyses—including time‐course experiments and studies in additional cell systems—are necessary to reach a firm conclusion, this interpretation is supported by reports showing that cycloheximide induces various mRNAs at the transcriptional level [54, 55, 56]. Exploring the function of YIPFα1A in stress responses under translational inhibition is an interesting direction for future research, because YIPFα1A has been reported to be involved in the unfolded protein response, in which translation is also suppressed [57, 58].

### Stabilization of YIPFα1A mRNA by the proximal region of 3′UTR

We found that the proximal fragment of the 3′ UTR (51–150) markedly stabilize YIPFα1A mRNA and leading to the increase in protein expression (Fig. 10). Regulation of mRNA stability and translation has been shown to depend on multiple *cis*‐regulatory elements embedded within the 3′ UTR [34, 37]. These elements can modulate mRNA abundance and final protein output by recruiting RNA‐binding proteins (RBPs) or microRNAs (miRNAs), which either enhance or repress gene expression. Therefore, it is likely that the proximal 3′ UTR fragment (51–150) contains functional *cis*‐regulatory elements that recruit such regulatory factors to stabilize the transcript.

Among RBPs, HuR is known to stabilize target transcripts by binding to AU‐rich elements within the 3′ UTR [59, 60]. Notably, the minimal stabilizing region of the YIPFα1A 3′ UTR (nt 51–150) contains an AUUUA motif near its center, which has been reported to mediate HuR binding. However, substituting the central U with G did not impair the stabilizing activity. Further analyses using additional mutations, along with protein pull‐down assays, will be necessary to elucidate the molecular mechanisms underlying mRNA stabilization mediated by this element.

### Reduction of protein expression by the distal region of 3′ UTR

The distal 3**′**‐UTR fragment (nt 1116–2230) did not increase protein levels, despite elevating mRNA to an extent comparable to the proximal fragment (nt 1–1118). Consequently, the protein‐to‐mRNA ratio (*fold* [protein]/*fold* [mRNA]) was reduced for distal‐fragment transcripts (~ 0.7, i.e., ~ 60% of proximal), indicating lower protein output per mRNA. To interpret these findings, we consider three questions.

(1) *Why does the distal fragment increase mRNA to a level similar to the proximal fragment?* Our mapping shows that the proximal region contains a stabilizing element (nt 51–150). A nonexclusive possibility is a length effect: longer 3**′** UTRs are generally associated with increased stability, which could explain the mRNA rise observed with both fragments. If this length‐dependent contribution does not add to the stabilizing activity of the proximal region, the overall outcomes can be accounted for.

(2) *What mechanism reduces protein output per mRNA with the distal fragment?* The simplest explanation, consistent with our observation, is translational suppression of distal‐bearing transcripts. This view is consistent with the higher fraction of degraded species in distal constructs (Fig. 8B, left panel), suggesting a pool of transcripts already committed to decay and thus poorly translated. Alternatively, the distal region may harbor a *cis*‐repressive motif/structure (e.g., RBP/miRNA‐mediated) that directly inhibits translation.

(3) *Why is reduced protein output not evident for the full‐length transcript?* Although mutation of the internal poly(A) site shifted isoform usage (larger isoform increased, smaller isoform decreased), protein levels remained unchanged (Fig. 5). This can be explained if the proximal 3**′**‐UTR region functionally overrides distal‐region repression in the full‐length context.

These possibilities should be tested directly using finer distal‐region deletions, swaps of distal and proximal 3**′**‐UTR segments, translation‐efficiency assays (e.g., polysome profiling or *in vitro* translation), and RBP/miRNA candidate screening.

### Protein expression control at the post‐translational level

When a construct containing the WT YIPFα1A CDS but lacking the 3′ UTR was used, the exogenously expressed mRNA level was approximately 20‐fold higher than the endogenous mRNA level (Fig. 2F,G). In contrast, exogenously expressed protein levels reached only about one‐eighth of the endogenous protein levels (Fig. 2A–C). Thus, the amount of protein produced per mRNA molecule was roughly 160‐fold higher for endogenous YIPFα1A mRNA than for exogenous mRNA lacking the 3′ UTR. Because addition of the full‐length 3′ UTR increased both mRNA and protein abundance to a similar extent (approximately 2.5‐fold; Fig. 4D), the presence of the 3′ UTR alone cannot account for the substantial difference in protein production efficiency per mRNA. These observations suggest that endogenous YIPFα1A expression is facilitated by an additional mechanism that enhances protein output relative to mRNA abundance (Fig. 2).

The major difference between endogenous and exogenous expression conditions appears to be the co‐expression (or lack thereof) of the *β*‐subunits (YIPFβ1A or YIPFβ1B). The absence of *β*‐subunit expression may impair translation of the exogenous mRNA or promote degradation of excess YIPFα1A protein lacking its partner subunit via the ER‐associated degradation (ERAD) pathway [61]. These possibilities are currently being investigated through co‐expression assays using YIPFα1A together with either YIPFβ1A or YIPFβ1B.

As discussed above, expression of the *β*‐subunits appears to be tightly coordinated with that of the *α*‐subunits. YIPF proteins form *α*/*β* heterodimers, and because each monomer contains five transmembrane segments, the final complex possesses 10 transmembrane domains. The *α*‐subunit must selectively interact with its cognate *β*‐subunit and avoid nonspecific interactions with other *β*‐subunits or unrelated membrane proteins. Therefore, it is reasonable to assume that complex assembly of the heterodimeric complex is precisely regulated during synthesis and processing in the ER. Nevertheless, the mechanisms and sub‐ER locations involved in membrane protein complex assembly remain poorly characterized.

In this context, studies reporting that 3′ UTRs regulate ER export of membrane proteins at a specialized ER subdomain—the TIGER domain [62, 63] provide an intriguing hypothesis. This model suggests that YIPFα1A synthesis, localization and subsequent trafficking may be regulated through interactions between the 3′ UTR and specific RBPs within a defined ER region, thereby promoting correct dimerization with *β*‐subunits. To test this possibility, we are developing methods to examine *in situ* mRNA localization together with the spatial distribution of the translated protein.

### Other possible control mechanisms

Although it is beyond the scope of this study, it should be noted that the 5′ UTR may also contribute to the regulation of YIPFα1A expression [64, 65]. Notably, the human YIPFα1A gene produces two mRNA isoforms that differ in the length of their 5′ UTR. The longer isoform (NM_0010297) contains 386 nts, whereas the shorter isoform (NM_030799) contains 135 nts in the 5′ UTR. The contribution of the 5′ UTR—either independently or in combination with the 3′ UTR—could be assessed in our experimental system by introducing or removing the 5′ UTR from reporter constructs.

### Physiological relevance of the multilayered control mechanism of YIPFα1A expression

Our findings have broader implications for understanding post‐transcriptional regulation in eukaryotic cells. The multilayered control of YIPFα1A expression highlights how cells coordinate the assembly of protein complexes, particularly membrane proteins that must avoid misfolding and aggregation. The conservation of suboptimal codon usage among human YIPF *α*‐subunits suggests that this feature represents an evolutionarily maintained regulatory mechanism rather than neutral drift. The slower translation kinetics resulting from suboptimal codon usage likely facilitates proper co‐translational folding and complex assembly while preventing protein aggregation [66]. These regulatory principles also have important practical implications for protein expression systems [67]. Our results indicate that successful heterologous expression of membrane protein complexes may require not only codon optimization strategies but also preservation of native regulatory elements, particularly 3′ UTRs that influence mRNA stability and translation efficiency.

The multilayered regulation of YIPFα1A expression also suggests that YIPF complex formation may be dynamically modulated under different physiological conditions, such as during development, cellular differentiation, or stress responses. This regulatory complexity likely reflects the necessity for precisely controlled YIPF complex stoichiometry, which is essential for maintaining Golgi architecture and overall cellular homeostasis. Future investigations into these regulatory networks will provide important insights into fundamental membrane trafficking processes and may shed light on disease states associated with Golgi dysfunction and aberrant YIPF protein activity.

## Conflict of interest

The authors declare no conflict of interest.

## Author contributions

NN conceived and supervised the research project. TT, YN, and NN conducted the experiments. NN prepared the manuscript.

## Data Availability

The data supporting the findings of this study are available from the corresponding author upon reasonable request.
